# Optimized methods for *Legionella pneumophila* release from its *Acanthamoeba* hosts

**DOI:** 10.1186/s12866-016-0691-x

**Published:** 2016-04-26

**Authors:** Elisabeth Dietersdorfer, Sílvia Cervero-Aragó, Regina Sommer, Alexander K. Kirschner, Julia Walochnik

**Affiliations:** Institute of Specific Prophylaxis and Tropical Medicine, Department of Medical Parasitology, Medical University of Vienna, Kinderspitalgasse 15, A-1090 Vienna, Austria; Institute for Hygiene and Applied Immunology, Water Hygiene, Medical University of Vienna, Kinderspitalgasse 15, A-1090 Vienna, Austria; Interuniversity Cooperation Centre for Water & Health, Vienna, Austria

**Keywords:** *L. pneumophila*, Endosymbiont, *Acanthamoeba*, Release

## Abstract

**Background:**

Free-living amoebae (FLA) and particularly acanthamoebae serve as vehicles and hosts for *Legionella pneumophila*, among other pathogenic microorganisms. Within the amoebae, *L. pneumophila* activates a complex regulatory pathway that enables the bacteria to resist amoebal digestion and to replicate. Moreover, the amoebae provide the bacteria protection against harsh environmental conditions and disinfectants commonly used in engineered water systems. To study this ecological relationship, co-culture and infection models have been used. However, there is a lack of data regarding the effectiveness of the different methods used to release intracellular bacteria from their amoebal hosts. The aim of this study was to evaluate the impact of the methods used to release intracellular *L. pneumophila* cells on the culturability of the bacteria. Furthermore, the standard method ISO 11731:1998 for the recovery and enumeration of *Legionella* from water samples was evaluated for its suitability to quantify intracellular bacteria.

**Results:**

The effectiveness of the eight release treatments applied to *L. pneumophila* and *Acanthamoeba* strains in a free-living state varied between bacterial strains. Moreover, the current study provides numerical data on the state of co-culture suspensions at different time points. The release treatments enhanced survival of both microorganisms in co-cultures of *L. pneumophila* and *Acanthamoeba*. Passage through a needle (21G, 27G) and centrifugation at 10,000 × g showed the highest bacterial counts when releasing the bacteria from the intracellular state. Regarding the ISO 11731:1998 method, one of the tested strains showed no differences between the recovery rates of associated and free-living *L. pneumophila*. However, a reduced bacterial recovery rate was observed for the second *L. pneumophila* strain used, and this difference is likely linked to the survival of the amoebae.

**Conclusions:**

Mechanical release treatments were the most effective methods for providing bacterial release without the use of chemicals that could compromise further study of the intracellular bacteria. The current results demonstrated that the recovery of *L. pneumophila* from water systems may be underestimated if protozoal membranes are not disrupted.

## Background

Free-living amoebae (FLA), particularly acanthamoebae, have recently gained scientific attention not only because of their intrinsic pathogenicity [1–3] but also because they serve as vehicles and hosts for a wide range of pathogenic microorganisms, such as *Coxiella burnetti*, *Chlamydophila pneumoniae*, *Mycobacterium* spp., *Pseudomonas aeruginosa*, *Vibrio cholera* and *Legionella pneumophila* [4–7].

Among these bacteria, the relationship between *Acanthamoeba* and *L. pneumophila* is one of the most studied due to the associated health risk. Legionellae are Gram-negative bacteria and common inhabitants of aquatic environments [8, 9]. Engineered habitats such as drinking water systems, hot water systems and cooling towers [10] may provide optimal conditions for the replication of these bacteria associated with protozoa. Legionellae are transmitted to humans via aerosols produced from these contaminated water sources and cause a severe lung infection called Legionnaires’ disease or a milder influenza-like form known as Pontiac Fever [11].

A wide range of disinfection techniques has been applied to control and prevent *Legionella* proliferation in drinking water systems [12]; however, cases of legionellosis may still occur. The protection from harsh environmental conditions and disinfectants that amoebae provide to intracellular bacteria has been studied in detail [13–16]. Furthermore, the molecular regulators that *L. pneumophila* uses at the transcriptional and (or) post-transcriptional level to control the expression of virulence traits and fitness factors to adapt to different intracellular (amoebae and macrophages) or extracellular environments have been revealed [17]. Additionally, various *L. pneumophila*-*Acanthamoeba* co-culture and infection models using chemical as well as mechanical methods to release the intracellular bacteria from their amoebal hosts have been described [18–26]. However, there is a lack of data regarding the different treatments and their effects on *L. pneumophila*.

The aim of this work was to establish a standard method for the release of intracellular *L. pneumophila* by testing and comparing several published and non-published protocols. Moreover, the effectiveness of the standard method commonly used for the isolation and quantification of *Legionella* spp. from water samples (ISO 11731:1998 [27]) was evaluated for its suitability for intracellular bacteria. Our results indicate that this method is prone to underestimating the number of surviving bacterial cells because *L. pneumophila* replicating vesicles within amoebae will grow as singles colonies on agar plates if the protozoal membranes are not disrupted. The current study provides optimised methods for the study of *Acanthamoeba-Legionella* interactions and of intracellular *L. pneumophila* from environmental samples.

## Results and discussion

There is a lack of information in the literature regarding the effectiveness of the methods used to release intracellular bacteria from amoeba hosts. In the current study, we assessed different approaches of release methods to determine the most appropriate method for each purpose.

### Effect of release treatments on pure cultures

To evaluate potential treatments for the release of intracellular bacteria, suspensions of two different *L. pneumophila* strains and two different *Acanthamoeba* strains were initially tested in a free-living state. Optimal treatments would result in a minimum log reduction of bacterial culturability but a high log reduction in amoebal culturability.

**Fig. 1:**
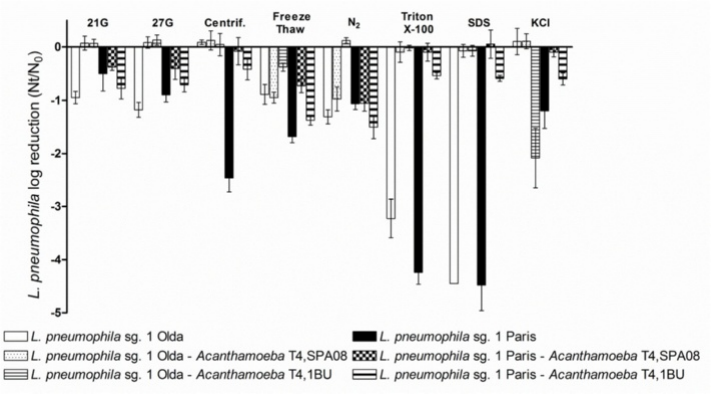
Effect of the eight release treatment methods applied to the free-living *L. pneumophila* strains and to co-cultures of the two *L. pneumophila* strains and the two *Acanthamoeba* strains. *L. pneumophila* inactivation was determined using viable counts on supplemented BCYE agar medium. Data are presented as the means ± SD (columns and error bars)

Among the eight treatments tested, mechanical treatments such as the passage through 21G and 27G needles, freezing-thawing cycles and treatment with liquid N_2_ resulted in a loss of approximately 1 log of cultivable *L. pneumophila* cells (Fig. 1). In contrast, chemical treatments using SDS and Triton™ X-100 reduced *L. pneumophila* culturability by 3–4.5 logs. That fact shows that the membrane composition of *L. pneumophila* particularly rich in branched fatty acids [28] is very sensitive to the effect of such detergents. Moreover, these results are in agreement with Moffat et al. [22], who noted that the use of Triton™ X-100 could damage *L. pneumophila* cells even at low concentrations. Interestingly, significant differences were observed between the two *L. pneumophila* strains tested. The *L. pneumophila* Paris strain was significantly more susceptible to treatment with Triton™ X-100 (*P* < 0.001), freezing-thawing (*P* < 0.01), centrifugation (*P* < 0.001) and KCl (*P* < 0.001) than the *L. pneumophila* Olda strain, particularly concerning the last two treatments. These results are in agreement with other authors who have already reported differences between *L. pneumophila* strains of the same serogroup towards drinking water disinfection methods [29]. Thus, preliminary tests should be performed on each *L. pneumophila* strain before choosing a release method.

**Fig. 2:**
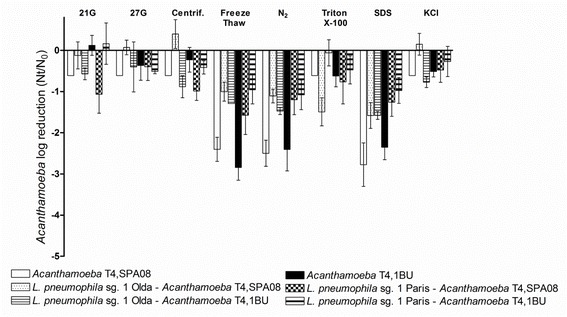
Effect of the eight release treatments methods applied to the free-living *Acanthamoeba* strains and to the co-cultures of the two *L. pneumophila* strains and the two *Acanthamoeba* strains. Amoeba inactivation was determined using the MPN method on NNA plates. Data are presented as the means ± SD (columns and error bars)

Regarding the amoebae, the chemical treatment with SDS and the mechanical treatments based on freezing-thawing and liquid N_2_ were the most effective; and reduced their culturability by 2–3 logs (Fig. 2). Although the resistance to a wide range of temperatures of some *Acanthamoeba* strains in a cystic live stage is well known [13, 30], the trophozoites of the two strains used were very sensitive to the thermal treatments applied. In contrast, the other release treatments reduced amoebal culturability by less than 1 log. No significant differences were observed between the two *Acanthamoeba* strains tested for any of the treatments applied (*P* > 0.05).

Because none of the treatments tested fulfilled the requirements to be considered optimal and due to the high variability between *L. pneumophila* strains, all eight treatments were tested also on the co-cultures of amoebae and bacteria.

### Intracellular growth of *L. pneumophila* within *Acanthamoeba*

**Fig. 3:**
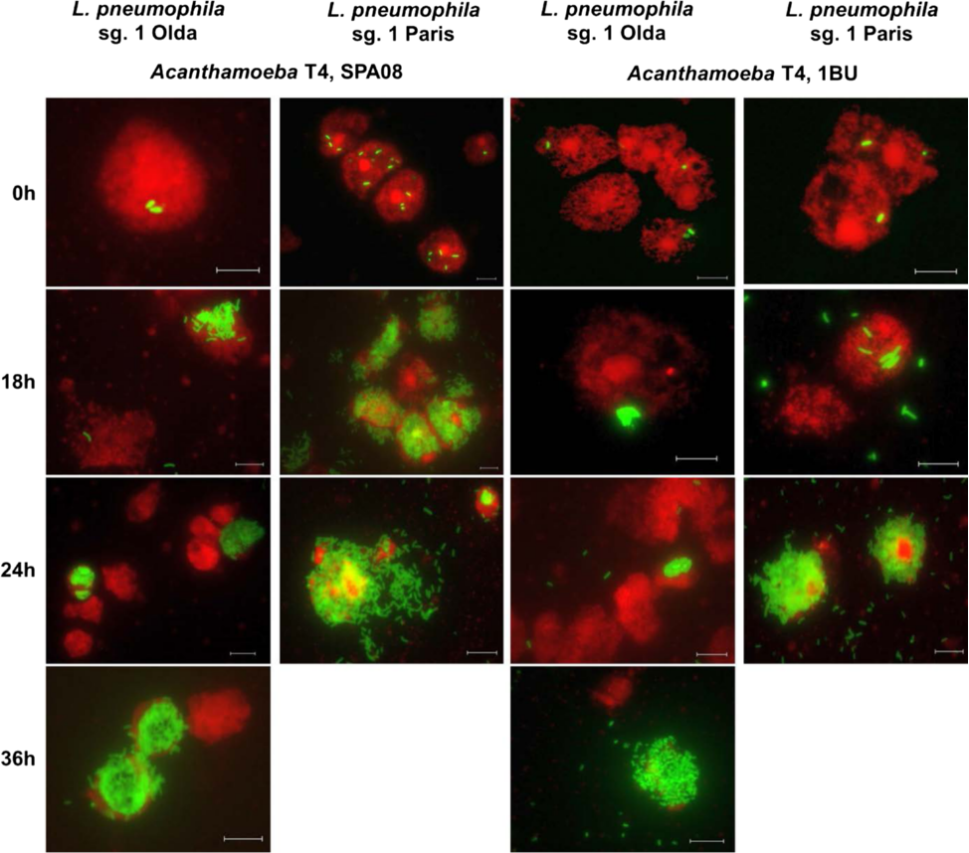
Intracellular growth of *L. pneumophila* strains within *Acanthamoeba* strains. Pictures were obtained using the MONOFLUO™ *Legionella pneumophila* IFA Test Kit to monitor the intracellular presence of *L. pneumophila* Paris (columns 2 and 4) and Olda (columns 1 and 3) within the *Acanthamoeba* strains SPA08 (columns 1 and 2) and 1BU (columns 3 and 4). The presence of *L. pneumophila* strains was determined at different time points: just after the co-culture preparation (T0) and at 18 h, 24 h and 48 h (T18, T24 and T36, respectively). Pictures were taken at 1000× magnification, scale bar = 9.2 μm

**Fig. 4:**
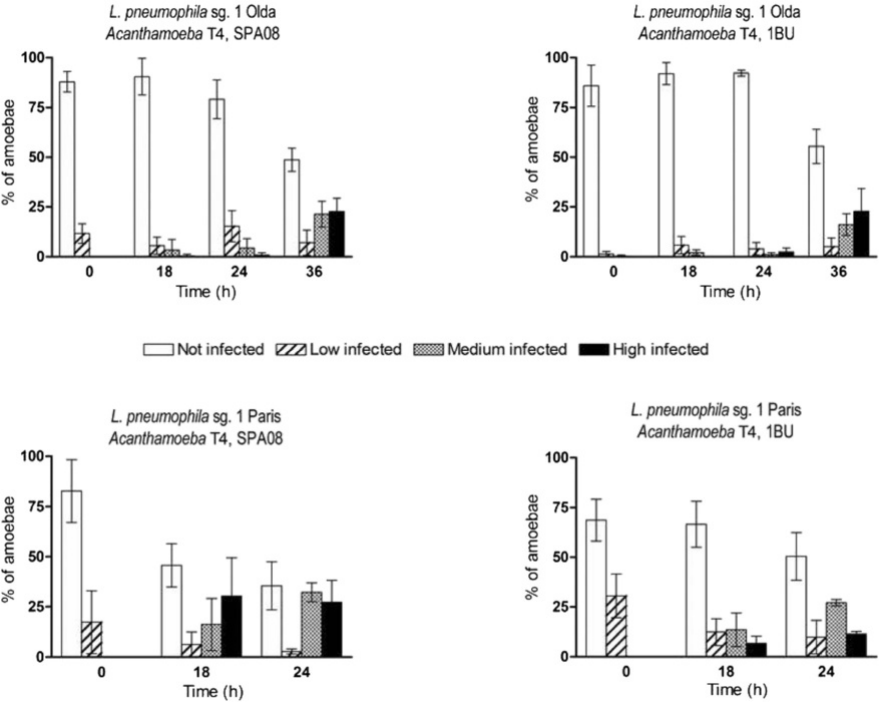
Efficiency of intracellular growth of *L. pneumophila* Paris and Olda within *Acanthamoeba* SPA08 and 1BU at different time points. Bacterial and amoebae cells were stained using the MONOFLUO™ *Legionella pneumophila* IFA Test Kit and counted using an epifluorescent microscope. Amoebae were divided in four different categories to describe their state: not-infected, low-infected (< 10 *L. pneumophila*/amoeba, early stage of infection), medium-infected (quantifiable replicating *L. pneumophila* cells) and high-infected (non-quantifiable, amoebae full of *L. pneumophila* cells with no cytosol left). Data are presented as the percentage ± SD (columns and error bars)

The intracellular growth of two *L. pneumophila* strains within two different *Acanthamoeba* strains was monitored using the MONOFLUO™ *Legionella pneumophila* IFA Test Kit (Fig. 3). The commercial kit consisted of FITC-labelled monoclonal antibodies that bind to the major outer membrane protein (MOMP) of *L. pneumophila* (in green) and a counterstain that labelled amoebal membranes (in red). This method resulted in a very useful, quick and easy manner of monitoring the bacterial state in co-culture suspensions. For the *L. pneumophila* Paris strain co-cultures, pictures were taken only for 24 h because after that time almost all of the bacterial cells were again in an extracellular state after bursting their amoebal hosts. All suspensions were analysed at different time points after co-culture and the number of infected and non-infected amoeba was assessed as well as the number of legionellae within every amoeba (Fig. 4). The state of the co-cultures in terms of the number of intracellular bacteria per amoeba was defined by using 4 different categories: 1. non-infected, 2. low-infected (< 10 *L. pneumophila*/amoeba, early stage of infection), 3. medium-infected (quantifiable replicating *L. pneumophila* cells), 4. high-infected (amoebae full of *L. pneumophila* cells, no cytosol left). According to the data obtained we determined the infectivity rate of each *L. pneumophila* strain, grouping categories 2, 3 and 4 versus category 1, and the time necessary to replicate in the amoeba host (Fig. 4). A similar approach to describe bacterial and amoebal interactions has been previously used [31].

The ability to replicate within amoeba hosts has been described by several authors as a strain-dependent event [29, 32]. The current study revealed that the extent of intracellular growth of *L. pneumophila* within *Acanthamoeba* indeed varies depending on both the bacterial and the amoebal strains used (Fig. 4). For example, amoebae of the category 4 with no cytosol left hosted around 40–50 bacterial cells whereas other amoebae hosted more than 100. The *L. pneumophila* Paris strain showed a higher virulence than the *L. pneumophila* Olda strain. This fact was represented first, by a higher infectivity rate, the *L. pneumophila* Paris strain infected a higher number of amoebal cells in 24 h, 62 ± 17 % in co-culture with the *Acanthamoeba* SPA08 strain and 49 ± 11 % in co-culture with the *Acanthamoeba* 1BU strain whereas, the *L. pneumophila* Olda strain infected 21 ± 13 % of the *Acanthamoeba* SPA08 strain and 8 ± 6 % of the *Acanthamoeba* 1BU strain at the same time point (Fig. 4). Second, *L. pneumophila* Paris strain was faster in replicating within the two *Acanthamoeba* hosts since after 18 h of co-culture 30 ± 19 % of *Acanthamoeba* SPA08 cells and 7 ± 3 % of *Acanthamoeba* 1BU cells were high-infected (category 4) (Fig. 4). However, it was necessary to prolong the co-cultures up to 36 h to observe more than 7 % of high-infected amoeba cells for the *L. pneumophila* Olda strain (Fig. 4).

The time point used in the release experiments was chosen according to the maximum number of amoeba infected and the maximum number of bacterial cell observed within them, category 3 and 4 (Fig. 4). Thus, treatments were applied after 24 h for the *L. pneumophila* Paris strain co-cultures and 36 h for the *L. pneumophila* Olda strain co-cultures.

### Effect of release treatments on co-cultures

To determine the best way to recover intracellular *L. pneumophila*, the eight release treatments were applied to the four co-cultures established.

The effectiveness of each treatment in releasing the intracellular bacteria was shown to depend on the respective strains (Fig. 1). Overall, the highest effectiveness, represented by the lowest loss of culturability, was observed in the *L. pneumophila* Olda co-cultures. The results of treatments such as passage through 21G and 27G needles and chemical treatments with SDS and Triton™ X-100 varied from 1 log to 4 log reduction in free-living bacteria suspensions to less than 0.5 log reduction or no reduction in co-culture for the *L. pneumophila* Olda strain. This fact confirms that most of *Acanthamoeba* membranes were disrupted by the release methods and beyond that *Acanthamoeba* cells play a protective role for *L. pneumophila* cells, as previously reported [14, 29]. Regarding the KCl treatment (*P* < 0.001) *L. pneumophila* Olda was divergently reduced depending on the *Acanthamoeba* co-culture. In this case, it was not clear whether the release treatment was not strong enough to disrupt amoebal membranes or in contrast with the studies by Berk et al. [18] and Holden et al. [33] the amoebal protection was not sufficient. Moreover, Barker et al. [28] reported changes in the membrane composition of intra-amoebic *L. pneumophila*, which could also explain some of the differences observed between intra- and extracellular states. There was a similar trend for the *L. pneumophila* Paris strain, although a higher variability between the treatments was observed (Fig. 1). Methods such as centrifugation, Triton™ X-100, SDS, freezing-thawing and KCl treatments resulted in a 2 to 4 log reduction of the culturability for *L. pneumophila* Paris suspensions but in less than 1 log reduction in co-culture with *Acanthamoeba*. As observed for the *L. pneumophila* Olda strain, the release treatment with liquid N_2_ was not sufficient to disrupt *Acanthamoeba* 1BU membranes. Comparing the two co-cultures of the *L. pneumophila* Paris strain, the highest loss of culturability was linked to the *Acanthamoeba* 1BU strain. Among the treatments applied, centrifugation and Triton™ X-100 treatments were the most effective for recovering cultivable cells. Interestingly, despite the harmful effect of Triton™ X-100 on free-living cells, this detergent has been used in many co-culture studies [13, 23, 34].

Regarding amoebal counts, it was shown that the effects of the release treatments tested were rather similar between the two *Acanthamoeba* strains (Fig. 2). The most effective methods, reducing amoeba counts by approximately 1.5 logs, were the treatments with SDS, freezing-thawing and liquid N_2_. However, these treatments also considerably reduced the culturability of *L. pneumophila*. Moreover, co-culture with *L. pneumophila* cells also reduced *Acanthamoeba* susceptibility to the treatments applied. For *Acanthamoeba* strain SPA08, the association with *L. pneumophila* Olda reduced the effect of centrifugation and liquid N_2_ whereas the association with *L. pneumophila* Paris reduced the effect of SDS and liquid N_2_. Moreover, for *Acanthamoeba* 1BU, the association with *L. pneumophila* Olda reduced the effect of freezing-thawing whereas the association with *L. pneumophila* Paris reduced the effect of treatment with SDS. Thus, co-culture between *L. pneumophila* and *Acanthamoeba* strains provides a reciprocal resistance to the applied treatments, as has been previously observed [34]. To understand the mechanisms of this reciprocal protection, further experiments are needed. In summary, the current study showed that mechanical release treatments such as passage through 21G and 27G needles as well as centrifugation can be considered optimal for the release of *L. pneumophila* from *Acanthamoeba* strains without the use of chemicals that could interfere with further experiments.

### Evaluation of the ISO 11731:1998 method in co-cultures

Suspensions of *L. pneumophila and Acanthamoeba* microorganisms in a free-living state and associated in co-culture were processed following the ISO 11731:1998 method for the detection and enumeration of *Legionella* [27].

**Fig. 5:**
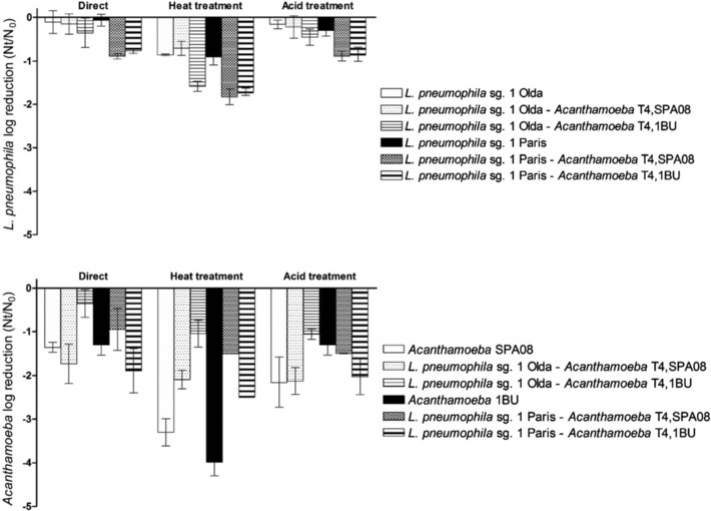
Effectiveness of the ISO 11731:1998 method in the recovery of *L. pneumophila* Paris and Olda and *Acanthamoeba* SPA08 and 1BU in a free-living state and in co-culture. Bacterial inactivation was determined using viable counts on supplemented BCYE agar medium, and amoebal inactivation was determined using the MPN method. Data are presented as the means ± SD (columns and error bars)

Despite the differences in the response to the release treatments between the two *L. pneumophila* strains as pure cultures, no significant differences (*P* > 0.05) were found in their recovery rates following the ISO method (Fig. 5). Direct plating and acid treatment resulted in a log reduction of culturability lower than 0.5 logs for the *L. pneumophila* strains, whereas heat treatment reduced culturability by almost 1 log. No significant differences (*P* > 0.05) were found in the recovery of non-associated *L. pneumophila* Olda and *L. pneumophila* Olda associated with the *Acanthamoeba* SPA08 strain for any of the methods analysed. However, the association with the *Acanthamoeba* 1BU strain, despite what was observed for most of the release treatments applied, significantly increased (*P* < 0.001) the susceptibility of both *L. pneumophila* strains to the heat treatment. The recovery of the *L. pneumophila* Paris strain notably varied depending on the bacterial state. Although no significant differences were found between the two co-cultures with *Acanthamoeba* strains, the recovery of the bacteria significantly decreased when compared with the free-living state for the direct plating (*P* < 0.001), heat treatment (*P* < 0.001) and acid treatment (*P* < 0.001) methods. This result could indicate that in the case of the *L. pneumophila* Paris strain, the standard method might not be harsh enough to disrupt the amoebal membranes. Thus, the replicating *L. pneumophila* vesicles from inside the amoebae grew as single colonies on the agar plates, resulting in an underestimation of the real number of bacterial cells. The analysis of amoebal recovery with the ISO method showed no significant differences (*P* > 0.05) between the two *Acanthamoeba* strains without bacteria (Fig. 5). The direct plating, heat treatment and acid treatment reduced amoeba culturability by approximately 1 log, 3.5 logs and 1.5 logs, respectively. The comparison between amoebae that were associated and those non-associated with *L. pneumophila* strains revealed that the association of *Acanthamoeba* SPA08 with the bacteria significantly reduced its susceptibility to the heat treatment (*P* < 0.001). In the case of *Acanthamoeba* 1BU, a similar behaviour was observed for the direct plating (*P* < 0.001) as well as for the heat treatment (*P* < 0.001). Thus, co-culture with bacteria enhanced amoebal survival. This phenomenon could explain the low recovery rates of the *L. pneumophila* Paris strain. Thus, depending on the *L. pneumophila* strains and their state, associated with amoebae or as free cells, their numbers could be significantly underestimated when using the ISO 11371:1998 method.

## Conclusions

In the current work, several release methods for the study of intracellular bacteria were assessed. Co-culture models were established using *L. pneumophila* and its amoebal host *Acanthamoeba*. Using an immunofluorescence assay specific for *Legionella pneumophila* proved to be extremely useful to monitor the intracellular state of the bacteria and to determine strain virulence. The investigation of the methods for intracellular *L. pneumophila* release from within *Acanthamoeba* host cells showed that mechanical release treatments, namely three passages through a 21G or 27G needle or centrifugation at 10,000 × g for 10 min, were the most effective treatments, showing the highest recovery rates without risk of interference by chemical residuals. When applying the ISO 11371:1998 method for the recovery of environmental *L. pneumophila* from water samples, the number of intracellular bacteria may be underestimated if the amoebal membranes are not destroyed during the detection procedure. The co-analysis of amoebal hosts such as acanthamoebae in positive samples for *L. pneumophila* could help to obtain more accurate results. To avoid the underestimation of *Legionella* spp. concentration in water samples, the inclusion of a mechanical pretreatment step (e.g., needle passage) in the ISO 11371:1998 enumeration method might be considered.

## Methods

### *L. pneumophila* strains

Release studies were conducted with 2 *Legionella pneumophila* SG1 strains. The type strain OLDA (ATCC43109) was provided by the AGES (Austrian Agency for Food and Health Security) strain collection, and the environmental isolate PARIS [35] was kindly donated by Y. Héchard, University of Poitiers. Strains were embedded in cryobeads (Roti®-Store Cryovials, Roth, Germany) and stored at −80 °C.

### *Acanthamoeba* strains

Experiments with amoebae were performed using 2 different *Acanthamoeba* genotype T4 strains 1BU (ATCC PRA-105) and SPA08 [36], both of which were isolated from keratitis patients. The strains were cultivated on non-nutrient-agar (NNA) plates previously seeded with heat-killed *E. coli* at room temperature (RT). From the NNA plates, axenic cultures were obtained according to the method described elsewhere [37]. Briefly, mature cysts were harvested from NNA plate cultures and incubated in 3 % HCl overnight to eliminate possible remaining bacteria. Suspensions were washed with Ringer solution 1/40 by centrifugation at 800 g for 15 min and transferred to 10 mL of PYG (proteose-peptone-yeast extract-glucose) medium (ATCC 712) media in 25 cm^3^ tissue culture flasks. Trophozoite cultures were maintained axenically by subculturing them in PYG in 25 cm^3^ culture flasks.

### Preparation of test suspensions and quantification of culturability after treatments

#### *L. pneumophila* suspensions

*L. pneumophila* strains were cultured on supplemented GVPC BCYE (buffered charcoal-yeast extract) agar (Biomerieux) at 37 °C for 72 h. Suspensions were prepared as described in Cervero-Aragó et al. [15]. Briefly, cells were harvested from the agar plates, resuspended in Ringer solution 1/40 and homogenized using a vortex mixer. The cell concentrations of the respective suspensions were adjusted by measuring the absorbance at 450 nm, using a photometer (Eppendorf Biophotometer). The concentrations of the tested *Legionella* suspensions were approximately 5 × 10^5^ cfu/mL. The treatments were applied as explained in Table 1.

**Table 1:** Description of the release treatments methods applied to *L. pneumophila* strains and *Acanthamoeba* strains in a free-living state or in co-culture

Release treatments methods	Description	Reference
Mechanical		
21 gauge needle (21G)	Passage three times through the needle	
27 gauge needle (27G)	Passage three times through the needle	[22, 31]
Centrifugation (Centrif.)	Centrifugation at 10,000 × g for 10 min	[21]
Freeze in liquid nitrogen (N_2_)	Immerse liquid N_2_ for 2 min and thawed at 35 °C for 10 min	[33]
Freezing-thawing cycles (Freeze Thaw.)	3 cycles of freezing-thawing consisting of 15 min at −80 °C followed by 10 min at 35 °C	[18]
Chemical		
Triton™ X-100	0.02 % Triton™ X-100 for 20 min at RT	[23]
SDS	0.5 % SDS for 10 min at rt	[24]
Salt treatment (KCl)	Centrifugation at 10,000 × g for 10 min, the supernatant was replaced with 1 mL of 0.038 M KCl, vortexed and incubated for 3 h at RT	[19]

#### *L. pneumophila* quantification after treatments

After each of the release treatments, ten-fold serial dilutions were prepared in Ringer solution 1/40 solution and transferred to supplemented BCYE plates for the enumeration of *Legionella* colony-forming units. The plates were incubated at 37 °C for up to 10 days and checked every 3–4 days.

#### *Acanthamoeba* suspensions

After the trophozoites had grown to confluence for 2–3 days at 30 °C in PYG medium, they were recovered from the tissue culture flasks with a soft shake and adjusted to a final concentration of 5 × 10^5^ amoeba cells/mL using a haemocytometer.

#### *Acanthamoeba* quantification after treatments

The concentration of viable of *Acanthamoeba* cells was quantified following the most probable number (MPN) method described elsewhere [38]. After the treatments ten-fold serial dilutions were prepared in Ringer solution 1/40 and transferred to NNA plates previously seeded with five spots of 20 μl of a fresh culture of *E.coli* Ten microliters of the diluted sample were added to these spots and plates were incubated at 30 °C for 8 days and checked every 2 days using an inverted microscope (Olympus CK2). The absence/presence of trophozoites in a dilution spot was considered negative/positive. The MPN values were obtained from MPN tables [39].

#### *Acanthamoeba*-*L. pneumophila* co-cultures

*Acanthamoeba* trophozoites were harvested by gently shaking the culture flasks to detach the cells and then transferring them to a 50 mL tube. Suspensions were counted as described above, and 5 × 10^5^ trophozoites were transferred to a 12-well cell culture plate. Trophozoites were incubated at 30 °C for 1 h to enable their adherence to the plate wells. After that, the PYG medium was replaced by a dilution 1:10 of PYG medium in Page's amoeba saline (PAS) (ATCC1323, [40]), and *L. pneumophila* suspensions were added at a ratio of 100 legionellae per amoeba. To enhance the interaction between legionellae and amoebae, plates were centrifuged at 500 × g for 10 min. After 2 h of incubation at 30 °C, the buffer was removed, and wells were washed twice with fresh and pre-warmed PAS buffer. To inactivate the remaining extracellular bacteria, co-culture suspensions were incubated for an hour at 30 °C with 50 ng/mL of gentamicin in the 1:10 PYG:PAS buffer. Then, wells were washed again, and fresh PYG:PAS buffer was added. This was denoted as time point 0. After evaluating several incubation times (see co-culture monitoring), incubation times of 36 h for *L. pneumophila* Olda and of 24 h for *L. pneumophila* Paris were chosen.

### Co-culture monitoring

*Acanthamoeba* co-cultures with *L. pneumophila* were analysed at different time points to determine the maximum possible number of *L. pneumophila* cells within an amoeba cell. This time point was considered the optimal time to apply the release methods. Briefly, 1 mL of the co-culture sample was washed twice by centrifugation at 1000 × g for 5 min by adding fresh phosphate-buffered saline (PBS). Then, 900 μL of the supernatant was discarded, and the pellet was resuspended in the remaining PBS. From this, 10 μL was placed onto a 10-well Teflon slide (Medco Health Solutions, Inc., Germany). Slides were incubated at 30 °C for 30 min to let the cells attach to the slide surface. The samples were fixed by incubation for 10 min at RT in 20 μL of 4 % paraformaldehyde (v/v PBS), washing once with PBS, and dehydrating for 3 min in an aqueous ethanol series (50, 80, and 96 %). Cells were then stained with the MONOFLUO™ *Legionella pneumophila* IFA Test Kit, which uses FITC-labelled monoclonal antibodies to detect the major outer membrane protein of *L. pneumophila*. Slides were then investigated using a Nikon Eclipse 8000 epifluorescence microscope, and photographs were processed with the software NIS Elements BR 2.3 (Nikon).

### Methods for the release of *L. pneumophila*

Both, mechanical and chemical methods (Table 1) were first applied to the different microorganisms in pure cultures and then to the respective co-cultures. One-millilitre suspensions of *L. pneumophila*, *Acanthamoeba* and the four co-cultures were prepared as explained above. After all treatments, the suspensions were placed in an ultrasonic bath (Bandelin Sonorex, RK 100, 35 kHz, Germany) for 2 min to enhance release of the bacterial cells.

### Evaluation of the ISO 11731:1998 method for the detection of intracellular *L. pneumophila*

All suspensions were processed as described in the ISO 11731:1998 for the detection and enumeration of *Legionella* [27]. Briefly, water samples were spiked with 10^5^ cells/mL in 100 mL of distilled water. After filtering the sample through a 0.22 μm polycarbonate filter, cells were recovered in 5 mL of Ringer solution 1/40 by sonication in an ultrasound bath for 2 min. Then, samples were divided into 3 aliquots. One aliquot was transferred to supplemented BCYE agar plates or NNA plates with *E. coli* in case of the amoebae without any further treatment. The second aliquot was transferred to the agar plates after being exposed to an acid buffer for 5 min, and the third aliquot was incubated at 50 °C for 30 min before transfer to the plates [27]. For every aliquot, ten-fold dilution series were made in Ringer solution 1/40 before transfer to the plates. Supplemented BCYE agar plates were incubated at 37 °C for up to 10 days and NNA plates were incubated at 30 °C for up to 7 days.

### Statistical analysis

The inactivation of different microorganisms was defined as a logarithmic reduction (N/N_0_), whereby N_0_ and N referred to the concentration of culturable cells of *L. pneumophila* or the MPN of amoebae before and after release treatments, respectively. The results are presented (or depicted) as the mean ± standard deviation (SD) and were plotted using GraphPad Prism 4. All data reported in this study were obtained from independent experiments performed in triplicate. The experimental conditions were statistically analysed using one-way ANOVA tests; *p* values less than 0.05 were considered to indicate statistical significances (GraphPad Software, San Diego, CA, USA). After the ANOVA test, a Bonferroni’s multiple comparison test was used to discern between the means in the case of significant differences (GraphPad Software, San Diego, CA, USA).

### Ethics approval and consent to participate

Not applicable.

### Consent for publication

Not applicable.

### Availability of data and materials

Data presented in this study are complete. No supplementary files are attached.

## Abbreviations

BCYE: buffered charcoal yeast extract
FITC: fluorescein isothiocyanate
FLA: free-living amoebae
GVPC: glycine, vancomycin, polymyxin b, cycloheximide
*L. pneumophila*: *Legionella pneumophila*
MOMP: major outer membrane protein
MPN: most probable number
NNA: non-nutrient agar
PAS: amoeba page’s saline
PBS: phosphate buffer
PYG: proteose-peptone-yeast extract-glucose
RT: room temperature
SD: standard deviation
